# Supplementary material for: Absolute Quantification of Pan-Cancer Plasma Proteomes Reveals Unique Signature in Multiple Myeloma
Source: Cancers (Basel). 2023 Sep 28;15(19):4764. doi: 10.3390/cancers15194764 (PMC10571728; doi:10.3390/cancers15194764)
Supplement: Supplementary file 1 [file cancers-15-04764-s001.zip › Supplementary Figure.pdf]

Supplementary Figures

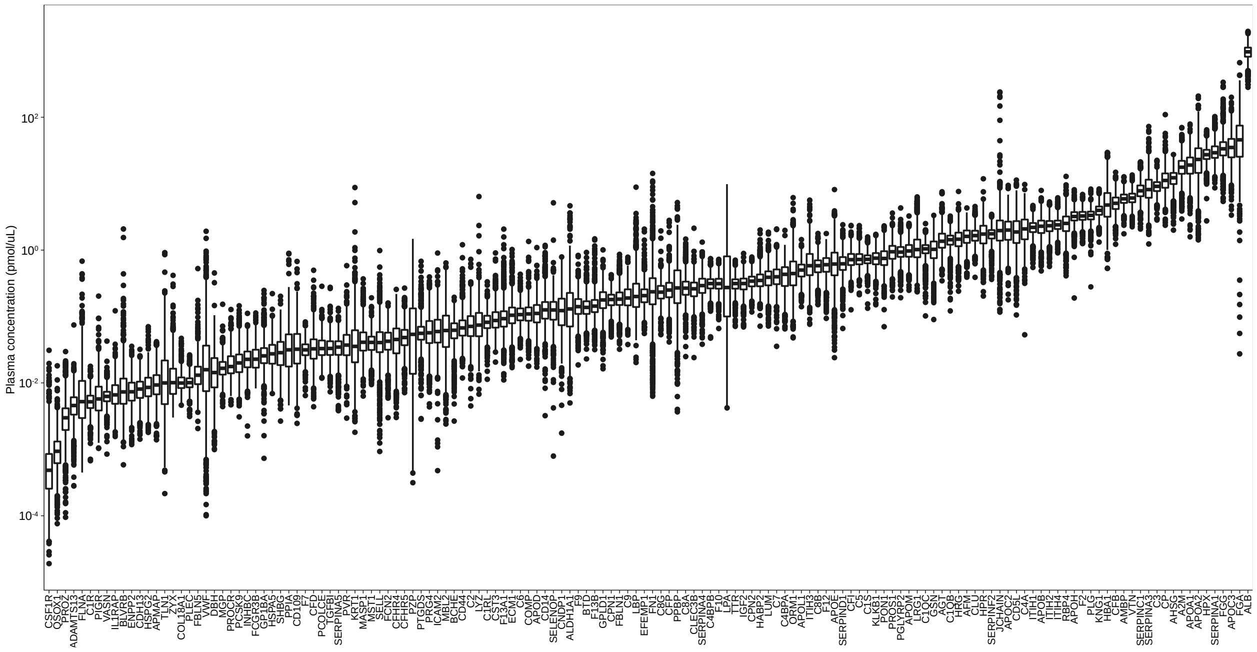

Figure S1 *– All 146 quantified proteins showing dynamic range over 6 orders of magnitude.*

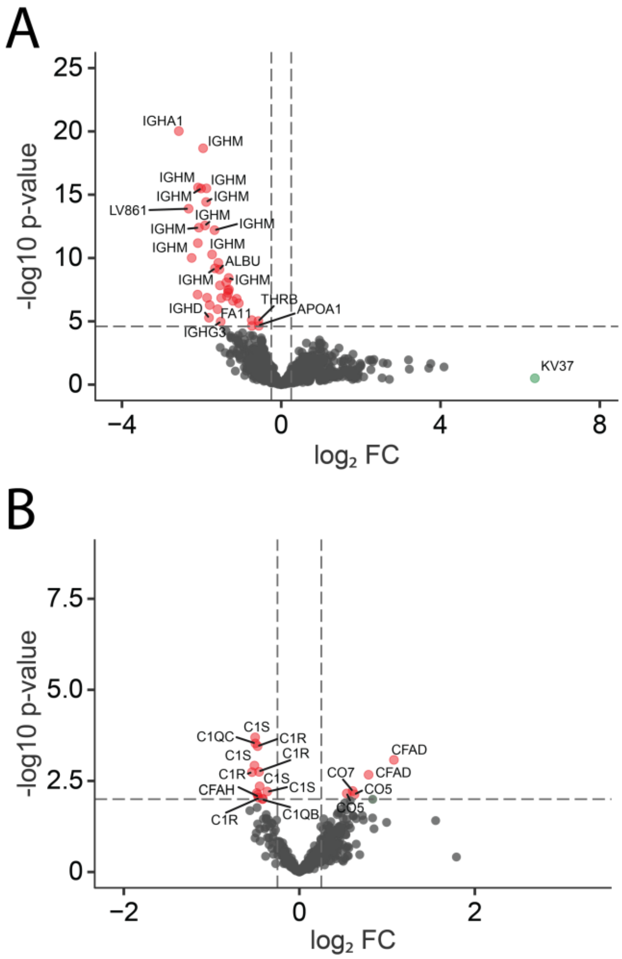

Figure S2 *– Label free quantification data showed protein dysregulation for multiple myeloma compared to all other cancers. A. Confirmed downregulated hallmark proteins for multiple myeloma linked to mainly*

immunoglobulins. B. Complement complex C1 proteins were observed downregulated in label free data, though, less significantly than in absolute quantification data.

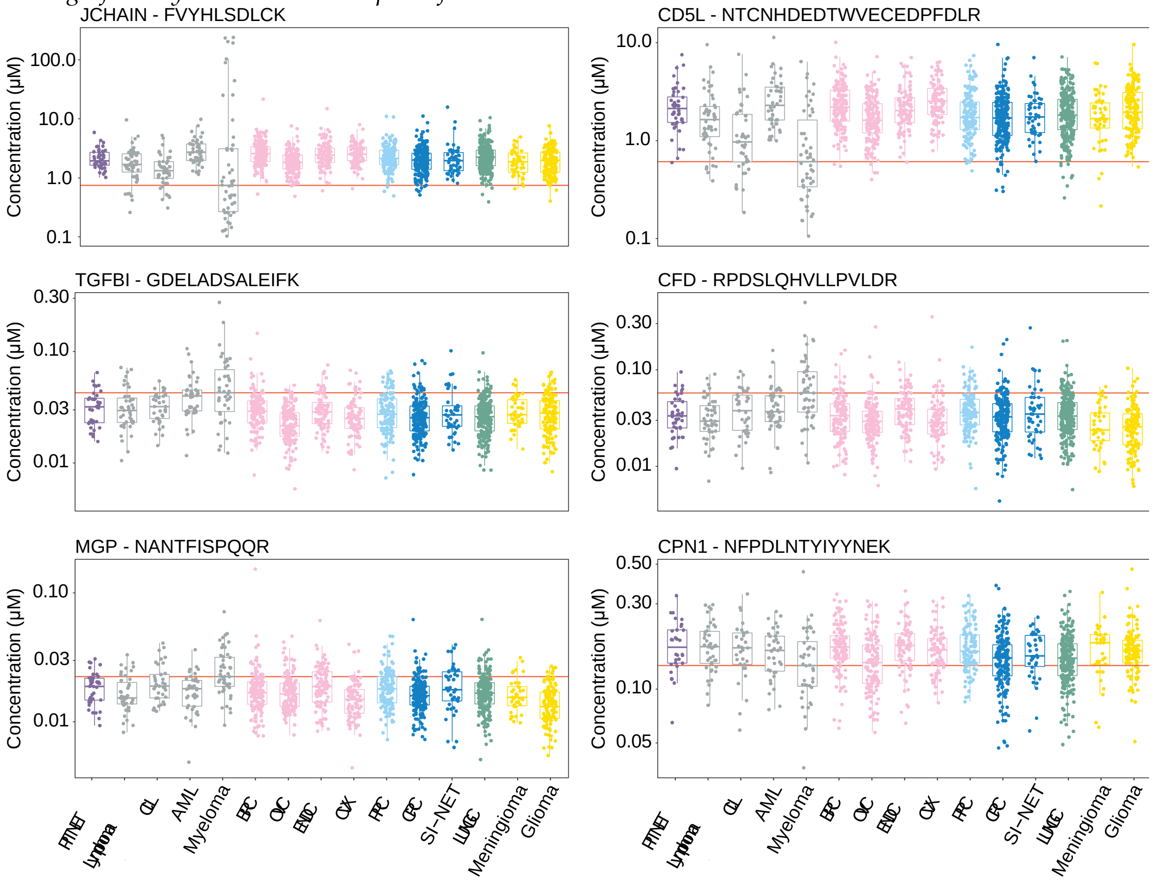

Figure S3 *– Peptides identified as the most important by the random forest models for the classification of multiple myeloma*

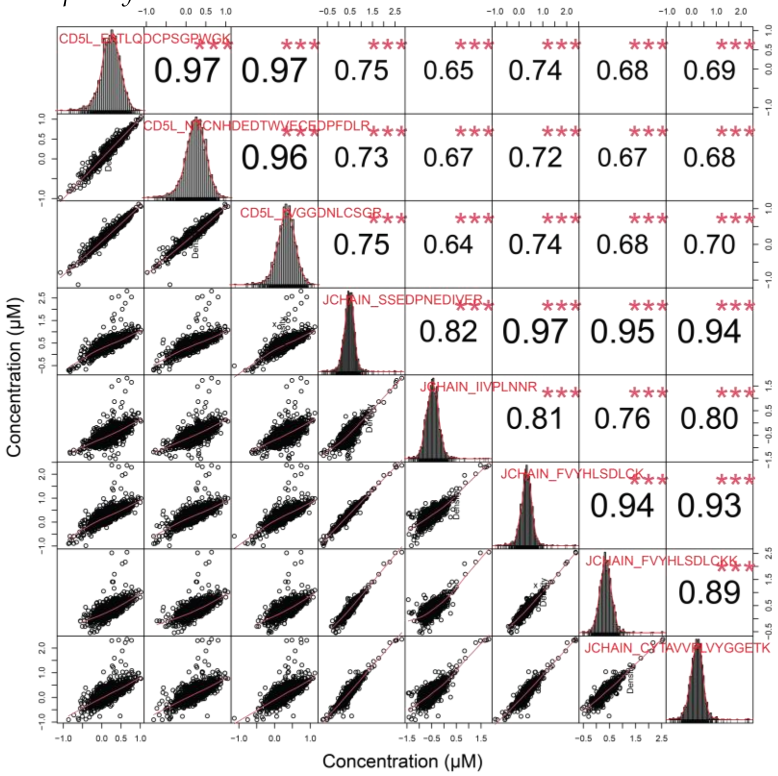

Figure S4 *– Correlation plot between all quantified peptides from CD5L and JCHAIN.*
